# Adaptation of *Cupriavidus metallidurans* CH34 to Toxic Zinc Concentrations Involves an Uncharacterized ABC-Type Transporter

**DOI:** 10.3390/microorganisms9020309

**Published:** 2021-02-02

**Authors:** Rob Van Houdt, Joachim Vandecraen, Natalie Leys, Pieter Monsieurs, Abram Aertsen

**Affiliations:** 1 Microbiology Unit, Interdisciplinary Biosciences, Belgian Nuclear Research Centre (SCK CEN), 2400 Mol, Belgium; joachim.vandecraen@sckcen.be (J.V.); nleys@sckcen.be (N.L.); pmonsieu@sckcen.be (P.M.); 2 Laboratory of Food Microbiology, Department of Microbial and Molecular Systems, Faculty of Bioscience Engineering, Katholieke Universiteit Leuven, 3000 Leuven, Belgium; abram.aertsen@kuleuven.be

**Keywords:** *Cupriavidus metallidurans*, zinc resistance, cadmium resistance, directed evolution, ABC transporter

## Abstract

*Cupriavidus metallidurans* CH34 is a well-studied metal-resistant β-proteobacterium and contains a battery of genes participating in metal metabolism and resistance. Here, we generated a mutant (CH34^ZnR^) adapted to high zinc concentrations in order to study how CH34 could adaptively further increase its resistance against this metal. Characterization of CH34^ZnR^ revealed that it was also more resistant to cadmium, and that it incurred seven insertion sequence-mediated mutations. Among these, an IS*1088* disruption of the *glpR* gene (encoding a DeoR-type transcriptional repressor) resulted in the constitutive expression of the neighboring ATP-binding cassette (ABC)-type transporter. GlpR and the adjacent ABC transporter are highly similar to the glycerol operon regulator and ATP-driven glycerol importer of *Rhizobium leguminosarum* bv. *viciae* VF39, respectively. Deletion of *glpR* or the ABC transporter and complementation of CH34^ZnR^ with the parental *glpR* gene further demonstrated that loss of GlpR function and concomitant derepression of the adjacent ABC transporter is pivotal for the observed resistance phenotype. Importantly, addition of glycerol, presumably by glycerol-mediated attenuation of GlpR activity, also promoted increased zinc and cadmium resistance in the parental CH34 strain. Upregulation of this ABC-type transporter is therefore proposed as a new adaptation route towards metal resistance.

## 1. Introduction

Metal homeostasis is important for all bacteria since they have to react swiftly to both the scarcity and excess of either essential or toxic metals [1,2,3]. To defend themselves against high metal toxicity, bacteria depend on multiple resistance mechanisms, including efflux pumps, proteins changing the oxidation state of metals, and intra- or extracellular sequestration of metals [4,5,6,7]. In addition, extracytoplasmic function (ECF) sigma factors play an important role in the response to environmental stressors as well as in metal homeostasis [8].

One of the essential metals is zinc, which occurs naturally in air, water, rocks, and soil. The average natural zinc level in the Earth’s crust is 70 mg/kg (dry weight), generally ranging between 10 and 300 mg/kg [9]. However, zinc has been concentrated to much higher levels at some locations, either by natural geological and chemical processes or through anthropogenic interventions because of the wide use of zinc compounds in industry, agriculture, and medicine [10,11,12,13,14,15,16]. Despite its essential role as a trace element in various biological processes, including proper functioning of specific enzymes, stabilization of DNA, and expression of genes [17], excess zinc has significant toxicity and acts as a potent disrupter of biological systems [18]. This duality of zinc properties requires a tight regulation of its intracellular homeostasis.

*Cupriavidus metallidurans* CH34 was one of the first bacteria to be isolated from industrial sites characterized by an extremely high metal content [19] and contains an unprecedented number of genes involved in the resistance and processing of metals [7]. *C. metallidurans* strains do not contain a high-affinity zinc uptake system like the ATP-binding cassette (ABC) uptake system ZnuABC from *Escherichia coli* [20]. Instead, uptake of zinc is accomplished by a set of highly redundant metal cation uptake systems with only minimal selectivity. In strain CH34, the only known import system with some specificity for zinc is ZupT [21,22], which is needed to deliver zinc under conditions of low availability [23]. The expression of *zupT* is upregulated under conditions of zinc starvation and repressed by FurC when sufficient zinc is present [24]. Deletion of *zupT* results in numerous defects caused by disturbed zinc homeostasis at lower and higher zinc concentrations [22].

*C. metallidurans* CH34 accomplishes metal detoxification by the concerted action of efflux systems, which may be followed by metal sequestration or complexation [25,26,27,28,29]. Various transporters remove excess zinc either from the cytoplasm or the periplasm. The most important zinc resistance operon is the *czc* cluster on megaplasmid pMOL30 [7,19,26,30,31]. The high-level metal resistance system Czc mediates the efflux of Co^2+^, Zn^2+^ and Cd^2+^, and loss of pMOL30 results in a drastically reduced zinc resistance [19]. The *czc* determinant is organized into two divergently transcribed gene clusters, i.e., *czcNICBADRSE* and *czcP*. The first cluster encodes CzcCBA belonging to the heavy metal efflux (HME)-Resistance-nodulation-division (RND)-driven efflux systems [32] and is comprised of three components spanning both outer and cytoplasmic membrane with an outer membrane protein (CzcC), a membrane fusion protein (CzcB), and a substrate-binding inner membrane transporter (CzcA) [33,34]. RND-driven efflux systems are responsible for the export of their substrates from the periplasm to the outside of the cell [35,36,37,38]. The *czcD* gene codes for a secondary transport system belonging to the Cation Diffusion Facilitator (CDF) family [27]. The second cluster codes for CzcP, a P_IB4_-type ATPase, which functions as a resistance enhancer exporting Zn^2+^ much more rapidly than P_IB2_-type ATPases, but it relies on the action of the latter to provide a basic resistance level [38]. The *czc* operon is zinc-inducible and under the control of the two-component regulatory system of CzcS (a histidine sensor kinase) and CzcR (a response regulator) [30,39]. However, a complex interplay exists between the plasmid-borne regions and chromosomal metal resistance clusters in zinc resistance. Two other efflux systems are inducible by zinc, i.e., the P_IB2_-type ATPases ZntA and CadA (both located on the chromosome), but their zinc induction is prevented in the presence of the *czc* operon [40]. Two more proteins of the CDF family, i.e., DmeF and FieF, which are both chromosomally encoded, were described to be mainly involved in cobalt and iron homeostasis, respectively, but have broad substrate spectrum and can probably also transport zinc [41]. In addition, *C. metallidurans* harbors a second RND transporter involved in zinc efflux, namely the chromosomally encoded Zne transporter [42,43]. Although this transporter is highly specific for zinc, it seems to have a dedicated function in zinc homeostasis and not in resistance to high zinc concentrations as the plasmid-free strain AE104 is zinc-sensitive [44]. The CDF and ATPase efflux systems transport their substrate from the cytoplasm to the periplasm, where RND-driven efflux systems will export these metals to the outside of the cell [8,27].

While the current zinc resistance mechanisms of CH34 have been well established, much less is known about its adaptive potential in the face of zinc stress. In this study we generated a mutant adapted to high zinc concentrations in order to shine a light on possible adaptive changes in CH34′s genome. As such, a novel determinant underlying increased zinc resistance could be identified.

## 2. Materials and Methods

### 2.1. Strains, Media, and Culture Conditions

The bacterial strains and plasmids used in this study are listed in Table 1 and Table 2. *C*. *metallidurans* was routinely cultured at 30 °C in Tris-buffered mineral medium (6.06 g/L Tris/HCl, 4.68 g/L NaCl, 1.49 g/L KCl, 1.07 g/L NH_4_Cl; 0.43 g/L Na_2_SO_4_, 0.2 g/L MgCl_2_·6H_2_0, 0.03 g/L CaCl_2_·2H_2_0, 0.04 g/L Na_2_HPO_4_·2H_2_O, 4.8 mg/L Fe(III)(NH4)citrate; 144 µg/L ZnSO_4_·7 H_2_O, 99 µg/L MnCl_2_·4H_2_O, 62 µg/L H_3_BO_3_, 190 µg/L CoCl_2_·6H_2_O, 17 µg/L CuCl_2_·2H_2_O, 24 µg/L NiCl_2_·6H_2_O, 36 µg/L Na_2_MoO_4_·2H_2_O) supplemented with 0.2% (*w*/*v*) sodium gluconate (MM284). *Escherichia coli* strains were routinely cultured at 37 °C in Lysogeny broth (LB). Liquid cultures were grown in the dark on a rotary shaker at 150 rpm. For culturing on agar plates, 2% (*w*/*v*) agar (Thermo Scientific, Oxoid) was added. When appropriate, the following chemicals (Sigma-Aldrich or Thermo Scientific) were added to the growth medium at the indicated final concentrations: kanamycin (50 µg/mL for *E. coli* (Km^50^) or 1500 µg/mL for *C. metallidurans* (Km^1500^)), tetracycline (20 µg/mL (Tc^20^)), chloramphenicol (30 µg/mL (Cm^30^)), Zn^2+^ (0.3, 12, 24, or 25 mM as zinc sulfate heptahydrate), Ni^2+^ (1.25, 2.5, or 5 mM as nickel chloride hexahydrate), Cd^2+^ (1, 1.5, 2, 3, or 4 mM as cadmium chloride hemipentahydrate), Co^2+^ (1.25, 2.5, or 5 mM as cobalt chloride hexahydrate), 5-bromo-4-chloro-3-indolyl-β-galactopyranoside (X-Gal; 40 µg/mL), and isopropyl β-D-1-thiogalactopyranoside (IPTG; 0.1 mM). Glycerol (Merck Millipore) was added to MM284 at a final concentration of 1% (*w*/*v*).

### 2.2. Isolation of Zinc-Resistant Mutants

*C. metallidurans* CH34 was first cultivated in MM284 at 30 °C until stationary phase and subsequently diluted 1:100 in MM284. Then Zn^2+^ was added to a final concentration of 12 mM. After four days of growth at 30 °C, 10^9^ cells were pelleted and cell suspensions (100 µL) of a serial tenfold dilution in saline (0.85% NaCl) were spread on MM284 agar plates containing a final concentration of 24 mM Zn^2+^ and incubated at 30 °C. Colony-forming units (CFU) were counted after day two and survival frequency was calculated as viable cell count on MM284 24 mM Zn^2+^ agar plates divided by viable cell count on MM284 agar plates.

### 2.3. Assessment of Zinc-Resistant Phenotype

The susceptibility of CH34 and CH34^ZnR^ to a metal ion were determined by the minimal inhibitory concentration (MIC). The strains were cultivated in biological triplicates by inoculating 2 mL MM284 or MM284 supplemented with different metals with 20 µL of a stationary phase *C. metallidurans* CH34 or CH34^ZnR^ culture. The MIC is defined as the lowest metal ion concentration that inhibits visible growth of the culture after two days of incubation at 30 °C.

In addition, the resistance of *C. metallidurans* strains was further assessed with dose–response growth curves, which were conducted in MM284 supplemented with different zinc or cadmium concentrations. Precultures were incubated at 30 °C until the stationary phase, diluted 1:100 in fresh medium with increasing zinc/cadmium concentrations, and incubated at 30 °C for 72 h. Next, the optical density at 600 nm (OD_600_) was determined in a 24-well cell culture plate (Flat-bottom, Greiner Bio-One, Vilvoorde, Belgium) which was placed into a CLARIOstar^®^ (BMG LABTECH, De Meern, The Netherlands). It is noteworthy that high Zn^2+^ concentrations resulted in precipitation (of zinc hydroxide) in an abiotic non-inoculated control, but did not impact optical density measurements (Figure S1).

To further analyze the phenotype of CH34^ZnR^, the cell survival of both CH34 and CH34^ZnR^ at a high zinc concentration (25 mM Zn^2+^) were determined. Stationary phase cultures were 1:100 diluted in fresh MM284 or MM284 supplemented with 0.3 mM Zn^2+^ (pre-induction assay) and these subcultures were allowed to grow for two days. Afterwards, 2-mL cell suspensions (biological triplicate) were transferred to a 24-well cell culture plate and Zn^2+^ was added to a final concentration of 25 mM. Next, 20 µL aliquots were withdrawn at different time points and cell suspensions (100 µL) of a serial dilution were spread on LB agar. The CFU/mL was determined after two days of incubation at 30 °C.

### 2.4. Construction of Plasmids

The *glpR* gene (Rmet_2235) from *C. metallidurans* CH34 and its zinc-resistant derivative CH34^ZnR^ were amplified by PCR (Phusion High-Fidelity DNA polymerase, Thermo Scientific, Aalst, Belgium) with primer pair Rmet_2235_Fw-Rv (Table S1), providing *Hin*dIII*/Eco*RI recognition sites. Afterwards, these PCR products were cloned as a *Hin*dIII*/Eco*RI fragment into pBBR1MCS2. The resulting pBBR-*glpR* and pBBR-*glpR*^R^ plasmids from *E. coli* DG1 transformants selected on LB Km^50^ were further confirmed by sequencing prior to conjugation (triparental with *E. coli* HB101 pRK600 as helper) to *C. metallidurans* CH34 and CH34^ZnR^.

### 2.5. Construction of Deletion Mutant Strains

The *glpR* gene (Rmet_2235) and the genes coding for the ABC-type transporter (Rmet_2229-2234) were amplified from *C. metallidurans* CH34 by PCR (Phusion High-Fidelity DNA polymerase) with primer pairs Rmet_2235_Fw-Rv and Rmet_2229_Fw-Rmet_2234_Rv (Table S1), respectively, providing *Hin*dIII*/Eco*RI recognition sites. Afterwards, these PCR products were cloned as a *Hin*dIII*/Eco*RI fragment into the mobilizable suicide vector pK18mob. The resulting p*glpR* and pRmet_2229-34 plasmids from *E. coli* DG1 transformants selected on LB Km^50^ were further confirmed by sequencing prior to amplifying of the flanking sequences of the R*glpR* or Rmet_2229-2234, respectively, by inverse PCR (Phusion High-Fidelity DNA polymerase) with primer pairs Rmet_2235_tet_Fw-Rv or Rmet_2229-2234_tet_Fw-Rv (Table S1), respectively, providing *Bcu*I*/Xba*I recognition sites. At the same time the *tet* gene from pACYC184 [46] was amplified by PCR (Phusion High-Fidelity DNA polymerase) with primer pair Tet_Fw-Rv (Table S1), providing *Bcu*I*/Xba*I recognition sites. Afterwards, this PCR product was cloned as a *Bcu*I*/Xba*I fragment into the former inverse PCR products. The resulting p*glpR::tet* and pRmet_2229-34*::tet* plasmids from *E. coli* DG1 transformants selected on LB Tc^20^Km^50^ were further confirmed by sequencing prior to conjugation (triparental with *E. coli* HB101 pRK600 as helper) to *C. metallidurans* CH34 or CH34^ZnR^. The resulting transformants selected on MM284 Tc^20^ were replica-plated on MM284 Tc^20^ and MM284 Km^1500^. CH34 ∆*glpR::tet* (CH34 ∆*glpR*), CH34 ∆Rmet_2229_34*::tet* (CH34 ∆29-34), and CH34^ZnR^ ∆Rmet_2229_34*::tet* (CH34^ZnR^ ∆29-34) cells resistant to Tc^20^ but sensitive to Km^1500^ were further confirmed by sequencing.

### 2.6. Whole-Genome Gene Expression Analysis and qRT-PCR

Whole-genome gene expression analysis of CH34 and CH34^ZnR^ was performed to examine which genes played a role in the increased zinc resistance. The strains were cultivated by inoculating 30 mL MM284 in biological triplicates with 300 µL of an exponentially growing *C. metallidurans* CH34 or CH34^ZnR^ culture at 30 °C. These subcultures were allowed to grow until an OD_600_ value of around 0.6 was reached. Next, each subculture was immediately subdivided in 15 microcentrifuge tubes of 2 mL and cells were harvested by centrifugation for 2 min at 10,000 rpm. Supernatant was removed and the bacterial pellets were flash frozen by immersion into liquid nitrogen and kept frozen at −80 °C at all times. RNA extraction, labeling and hybridization, microarray spotting, scanning, and data analysis were performed according to the work of [48]. The full description of the microarray data has been deposited at the Gene Expression Omnibus website (http://www.ncbi.nlm.nih.gov/geo/) under accession number GSE156826.

In addition, total RNA was extracted from CH34, CH34 Δ*glpR*, and CH34^ZnR^ (similar conditions as above) and single-stranded complementary DNA (cDNA) was synthesized from 1 μg total RNA using random hexamers as primers and the TaqMan Reverse Transcription Reagents (Thermo Scientific). The quantity of synthesized cDNA was measured with a NanoDrop^®^ 2000 spectrophotometer (Thermo Scientific). The expression of Rmet_2229 was analyzed by qRT-PCR using the QuantiNova SYBR Green RT-PCR kit (Qiagen, Venlo, The Netherlands) according to the manufacturer’s protocol with primers qF_2229 and qR_2229. Expression was compared with that of the 16S rRNA gene (Table S1). RT-qPCRs were performed with a Rotor-Gene Q (Qiagen).

### 2.7. Genome Sequencing

Whole-genome sequencing was performed to identify mutations responsible for the increased zinc-resistance of CH34^ZnR^. The strain was cultivated by inoculating 4 mL LB at 30 °C, and total DNA was extracted using the QIAamp DNA Mini Kit (Qiagen). The quantity and quality of extracted DNA was measured using a NanoDrop^TM^ 1000 spectrophotometer (Thermo Scientific). Ten micrograms of DNA were sent for Illumina sequencing (Baseclear, Leiden, The Netherlands). Large insertions and deletions (>200 bp) were identified using an in-house developed software able to exploit the paired-end characteristics of the sequencing data, where either an unexpected increase of unpaired sequencing reads was used as an indicator for insertions, and an increase in the distribution of insert sizes interpreted as deletion. The Genome Analysis Toolikt (GATK) was used to identify point mutations, i.e., single-nucleotide polymorphisms (SNPs) and small indels [49,50]. Sequencing data are available within the Sequencing Read Archive (SRA) of NCBI using the accession number PRJNA658861.

## 3. Results

### 3.1. Isolation and Characterization of Zinc-Resistant CH34 Derivatives

Direct exposure of *C. metallidurans* CH34 to a high Zn^2+^ concentration (24 mM Zn^2+^) is bactericidal, as no survivors were observed even after prolonged incubation (over two weeks). However, CH34 derivatives could be isolated on MM284 agar plates with 24 mM Zn^2+^ when it was first cultivated in liquid MM284 medium with 12 mM Zn^2+^ (i.e., parental MIC). Growth in liquid MM284 medium with 12 mM Zn^2+^ was observed after four days of incubation (no visible growth after two days of incubation) and subsequent plating of this culture on MM284 agar plates with 24 mM Zn^2+^ yielded zinc-resistant CH34 mutants at a frequency of 2.37 ± 0.30 × 10^−7^ (calculated after two days of incubation at 30 °C). One mutant was further purified on MM284 and retested for its resistance. The latter, designated as CH34^ZnR^, exhibited an inheritable resistance phenotype and was further characterized phenotypically. Compared to its parental strain, CH34^ZnR^ exhibited a two-fold increased resistance to both Zn^2+^ and Cd^2+^. No increased resistance to Ni^2+^ and Co^2+^ was observed (Table 3), indicating that the involved resistance mechanism(s) can detoxify both Zn^2+^ and Cd^2+^.

**Figure 1:**
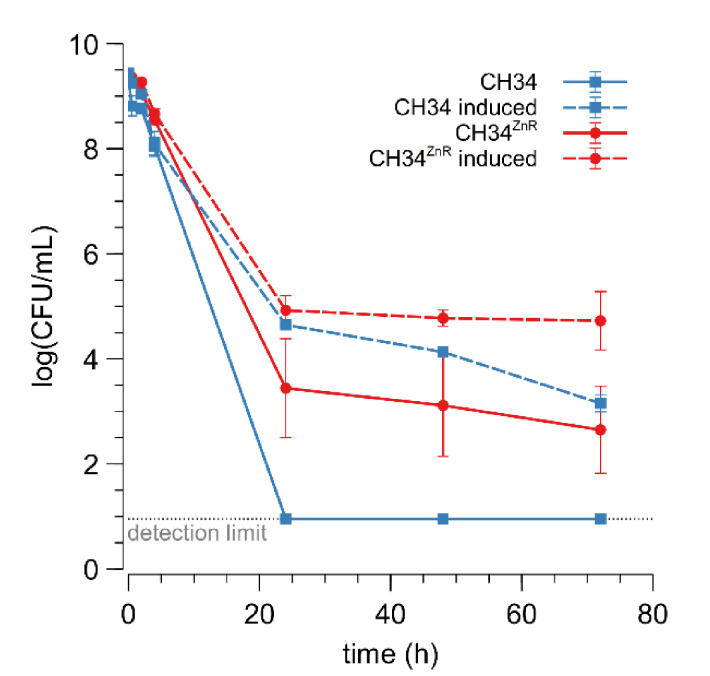
Cell survival upon exposure to 25 mM Zn^2+^ of *C. metallidurans* CH34 (blue square) and CH34^ZnR^ (red circles) without (full) or with (dashed) pre-induction with 300 µM Zn^2+^ for 48 h. The light grey dotted line represents the detection limit. The average values of three independent experiments with standard deviations are shown. CFU: colony forming unit.

In addition to MIC determination, inactivation dynamics in the presence of a high zinc concentration (25 mM) were examined for the parent and its CH34^ZnR^ derivative (Figure 1). The fraction of surviving CH34 cells drastically decreased in the presence of 25 mM Zn^2+^ and no survivors could be detected after 24 h of incubation. Although a drastically decreased cell survival in the first hours of incubation was also shown for CH34^ZnR^, a fraction of the cells did survive (2.85 ± 2.07 × 10^−4^ CFU/mL after 24 h). Pre-inducing the *czc* operon with 0.3 mM Zn^2+^ for 48 h [30,51,52] resulted in an increased cell survival of both the parental and zinc-resistant derivative (Figure 1). This derepression enables a fraction of the wild type cells to survive a very high concentration of Zn^2+^ because the Czc efflux pumps are already active before the cell encounters the toxic Zn^2+^ concentration [30,51,52]. However, cell survival for pre-induced CH34^ZnR^ was still higher than for pre-induced CH34. These observations suggest that the mutation(s) in CH34^ZnR^ do not target the *czc* operon and that its genetic background works synergistically with a zinc-induced *czc* operon, leading to more cells that survive.

### 3.2. Whole-Genome Expression Profile and Genome Sequence Analysis of CH34^ZnR^

**Figure 2:**
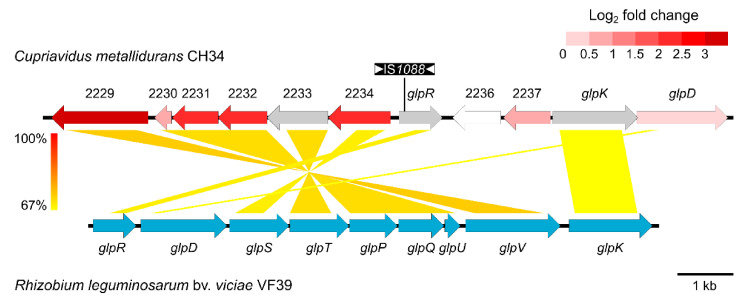
BLASTn comparison of the ABC-type transporter of *C. metallidurans* CH34/CH34^ZnR^ and the glycerol utilization cluster of the plasmid pRleVF39c from *R. leguminosarum* bv. *viciae* VF39 (accession no. JN390944). The colored shading indicates nucleotide identity between the sequences. Coding sequences (CDSs) of *C. metallidurans* CH34 are color-coded based on their log2 fold change (CH34^ZnR^ vs. CH34 expression), with grey being not detected. Insertion of IS*1088* in *glpR* of CH34^ZnR^ is shown.

As the increased resistance stemmed from natural selection of resistance-conferring mutations [53], the global shift in transcriptome resulting from the altered genotype of the evolved strain (CH34^ZnR^) as compared to the parental strain was examined in non-selective conditions [54,55,56]. This revealed 61 coding sequences (CDSs) that were significantly differentially expressed in comparison with the parental CH34 (>1 log_2_ fold with an adjusted *p*-value < 0.05), of which 21 were upregulated and 40 downregulated (Table S2). The ABC-type transporter (Rmet_2229_2234) (Figure 2) and the flagellar filament structural protein (*fliC2*) were the most upregulated genes. The *cupRAC* operon was the most downregulated, with *copK* and *copM* also being downregulated, but no alteration in copper resistance was observed (data not shown). Interestingly, no overlap was observed between the transcriptional profile of CH34^ZnR^ in non-selective conditions and the previously established transcriptional response of the parental CH34 strain to zinc stress [48].

Subsequent whole genome sequencing of strain CH34^ZnR^ revealed seven mutations, all of which were caused by transposition of insertion sequences (IS), more specifically IS*Rme5*, IS*Rme15* and IS*1088* (Table 4). Taking into account the expression profile, the inactivation of Rmet_2235 (*glpR*) by insertion of IS*1088* stands out. Therefore, we hypothesized that inactivation of Rmet_2235 resulted in derepressed transcription of the adjacently encoded ABC-type transporter (Rmet_2229-2234) and subsequent increased zinc and cadmium tolerance (Figure 2). This derepression was confirmed by qRT-PCR as transcription of Rmet_2229 was increased 3-fold in CH34 Δ*glpR* compared to the parental CH34 strain (Figure S2). Existing tagRNA-seq data used to identify the 5′ ends of RNAs in *C. metallidurans* CH34 corroborated that Rmet_2229-2234 was an operon transcribed from the same promoter into one polycistronic mRNA [57]. Furthermore, this dataset indicated that the main transcription start site of the *glpR* gene (for strain CH34 in non-selective growth conditions) was found 9 bp downstream of the currently annotated start codon, indicating translation of a 3-aa shorter GlpR from a leaderless transcript (Figure S3).

A similar gene cluster, also containing a glycerol kinase (*glpK*) and glycerol 3-phosphate dehydrogenase (*glpD*), was detected in various α-proteobacteria enabling utilization of glycerol as a carbon source [58]. In fact, the cluster is similar to the plasmid-borne locus responsible for glycerol utilization from plasmid pRleVF39c in *Rhizobium leguminosarum* bv. *viciae* VF39 [58]. In this cluster, the *glpR* gene codes for the repressor of the glycerol-3-phosphate regulon and negatively regulates the adjacent genes involved in glycerol metabolism.

### 3.3. ABC-Type Transporter (Rmet_2229-2234) Is Responsible for Increased Zinc Resistance

**Figure 3:**
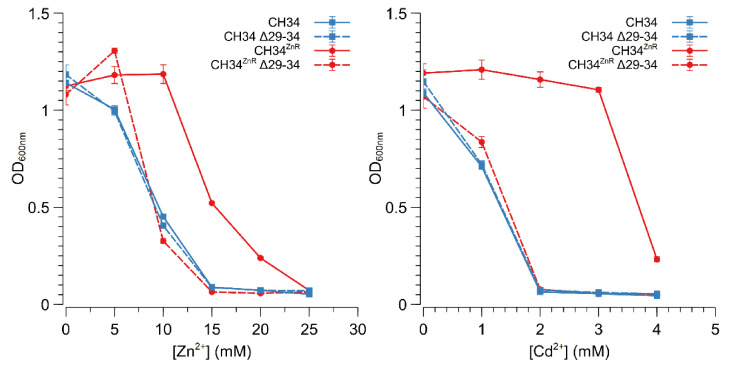
Zinc and cadmium resistance of *C. metallidurans* CH34 and its indicated derivatives. Zinc (left) and cadmium (right) dose–response experiments with CH34 (blue full line, squares), CH34^ZnR^ (red full line, circles) and their Δ29-34 derivatives (blue dashed line with squares and red dashed line with circles, respectively). The average values of three independent experiments with standard deviations are shown.

**Figure 4:**
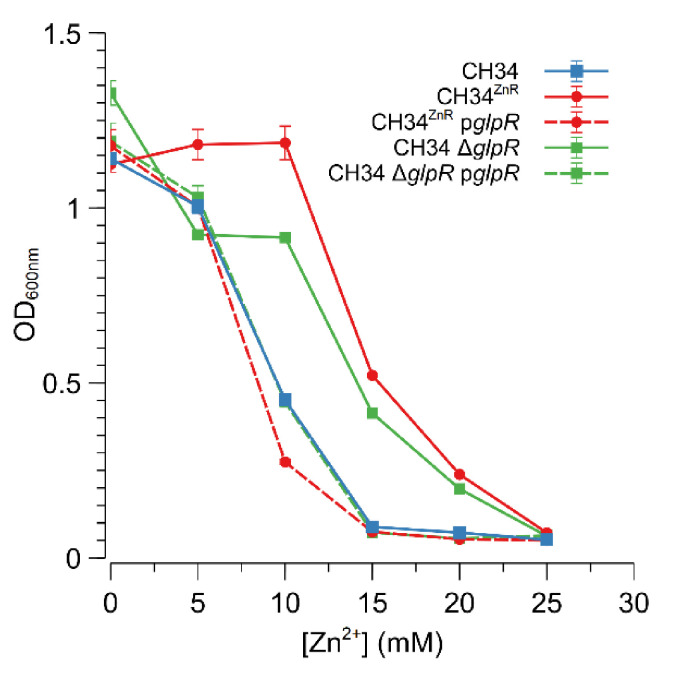
Impact of GlpR function on the increased zinc resistance of *C. metallidurans* CH34. Dose–response experiments with CH34 Δ*glpR* (green, squares) and CH34^ZnR^ (red, circles) without (full lines) or with (dashed lines) plasmid-based *glpR* complementation. The dose–response experiment with the parental CH34 strain is shown as a blue line (squares). The average values of three independent experiments with standard deviations are shown.

The Rmet_2229_2234 gene cluster was subsequently deleted by replacing it with a tetracycline resistance cassette (referred to as ∆29-34) in both CH34 and CH34^ZnR^ to test the hypothesis that constitutive derepression of the ABC-type transporter is responsible for CH34^ZnR^’s increased zinc resistance. First of all, no differences in zinc and cadmium resistance could be observed between CH34 ∆29-34 and its parental strain, indicating that the native ABC-type transporter exerts no role in the detoxification of zinc or cadmium in the parental CH34 strain (Figure 3). However, when the Rmet_2229-2234 gene cluster was deleted in CH34^ZnR^, the zinc and cadmium resistance of the resulting CH34^ZnR^ ∆29-34 mutant dropped to levels similar to those of the parental CH34, indicating that the ABC-type transporter is required for its increased zinc and cadmium resistance (Figure 3). In turn, deletion of the *glpR* gene in CH34 resulted in increased zinc resistance, while plasmid-based complementation of *glpR* reduced zinc resistance back to the parental level in both CH34 Δ*glpR* and CH34^ZnR^ (Figure 4). The latter further confirms that loss of GlpR function results in increased zinc resistance.

Finally, CH34 ∆29-34 was still able to utilize glycerol as a sole carbon source (data not shown), demonstrating that the ABC-type transporter is not essential for glycerol uptake in *C. metallidurans* (contrary to *R*. *leguminosarum* bv. *viciae* VF39).

### 3.4. Glycerol Induces Increased Zinc Resistance

**Figure 5:**
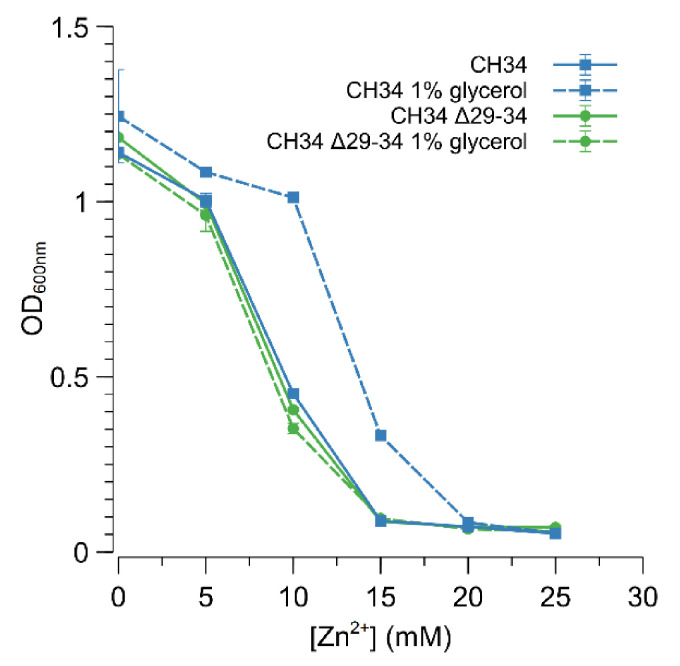
Impact of glycerol on the increased zinc resistance of *C. metallidurans* CH34. Dose–response experiments with CH34 (blue) and CH34 Δ29-34 (green) without (full line) and with (dashed line) 1% glycerol. The average values of three independent experiments with standard deviations are shown.

In *E. coli*, direct interaction with glycerol or glycerol-3-phosphate impedes the action of the GlpR repressor, and thus relieves the repression of the glycerol metabolism [59]. Therefore, *C. metallidurans* CH34 and CH34 ∆29-34 were grown in MM284 containing 1% (*w*/*v*) glycerol to examine if pre-induction with glycerol could promote derepression of the ABC-type transporter and consequently result in increased zinc resistance. Indeed, CH34 displayed a higher resistance to zinc in the continued presence of glycerol (Figure 5), putatively indicating that binding of GlpR to the operator of the ABC-type transporter is diminished in the presence of glycerol. Furthermore, glycerol had no direct effect on zinc resistance as no increase in zinc resistance was observed for CH34 ∆29-34 (Figure 5). However, a lower level of zinc resistance was reached for glycerol-induced CH34 compared to CH34^ZnR^ or CH34 ∆*glpR*, respectively, suggesting that the presence of glycerol was not sufficient to bind with all available GlpR repressors or that glycerol–GlpR interaction allowed a reduced repression of the ABC-type transporter.

## 4. Discussion

This study presents evidence that *C. metallidurans* CH34 can readily improve its extreme zinc and cadmium resistance through adaptive evolution. It is noteworthy that the high Zn^2+^ concentrations used resulted in precipitation (of zinc hydroxide) in an abiotic non-inoculated control. Therefore, in the presence of cells there will be a dynamic equilibrium between free Zn^2+^ ions, protein-bound Zn^2+^, and precipitation. As correct measurement of free Zn^2+^ is not straightforward [60], such analyses were out of the scope of this study. However, even without these analyses, the central observation of this study, which is the significant and consequent growth difference between the parental and adapted strain in the presence of high zinc concentrations, remains valid. Moreover, whole genome sequencing, expression profiling, and genetic analysis unexpectedly revealed that insertion sequence-mediated inactivation of the GlpR transcriptional repressor and subsequent upregulation of a neighboring ABC transporter (not previously involved in metal resistance) were causally responsible for the resistance phenotype.

GlpR belongs to the DeoR-family of transcriptional regulators that are widespread in bacteria and commonly function as specific regulators of carbon source uptake and catabolism, often playing a role in catabolite repression [59,61]. It shares 46% and 47% amino acid identity with the GlpR repressor of the glycerol 3-phosphate regulon in *E. coli* and the glycerol regulon in *R*. *leguminosarum* bv. *viciae* VF39, respectively. Glycerol uptake in *R*. *leguminosarum* bv. *viciae* VF39 is mediated by ABC-type active transport, in contrast to the GlpF-mediated facilitated diffusion in *E. coli* [62]. Uptake is followed by the enzymatic activity of glycerol kinase (*glpK*) and glycerol-3-phosphate dehydrogenase (*glpD*), for which expression is negatively regulated by GlpR binding to their operator sequences, enabling the use of glycerol as sole carbon source. In *E. coli*, *glpD*, but not *glpK*, is located near *glpR*. In contrast, in *R*. *leguminosarum* bv. *viciae* VF39, *glpD* and *glpK* flank the *glpSTPQUV* genes coding for an ABC transporter and form (together with the directly upstream located *glpR*) its plasmid-borne *glp* operon [58]. In both *E. coli* and *R*. *leguminosarum*, the glycerol utilization regulon is inducible by glycerol and glycerol-3-phosphate via the specific and direct interaction of these metabolites with the GlpR repressor, thereby causing derepression of the *glp* genes [58,62]. In *C. metallidurans* CH34, *glpK* (Rmet_2238) and *glpD* (Rmet_2239) are found in close proximity to *glpR* and to the genes coding for the ABC-type transporter (Rmet_2229-2234), although they do not form an operon with the transporter, reflecting a different genomic arrangement than in *R*. *leguminosarum* bv. v*iciae* VF39 (Figure 2). Only a slight differential expression of *glpD* (1.3-fold) was detected in CH34^ZnR^ (expression of *glpK* could not be determined). As *glpK* (Rmet_2238) and *glpD* (Rmet_2239) likely form an operon (transcription start sites not detected by the existing tagRNA-seq data [57]), it is conceivable that expression of *glpK* is also only slightly affected in CH34^ZnR^. In contrast to the derepression of the ABC-type transporter (Rmet_2229-2234), these data do not univocally show GlpR-mediated regulation of *glpKD* (Rmet_2238-39) in *C. metallidurans*. In addition, CH34 carries a second locus containing a *glpK* (Rmet_5445) and *glpD* (Rmet_5444) gene, as well as genes coding for a putative glycerol uptake operon antiterminator regulatory protein (Rmet_5442), a putative akylglycerone-phosphate synthase (Rmet_5443), and a Major Facilitator Superfamily (MFS) transporter (Rmet_5446). However, none of the genes in this second *glp* locus are differentially expressed in CH34^ZnR^. Furthermore, as glycerol can still be used as sole carbon source in the absence of the ABC-type transporter (CH34 Δ29-34), other glycerol transporters exist, e.g., the MFS transporter in the second *glp* locus (Rmet_5446).

The ABC-type transporter responsible for increased zinc and cadmium resistance in CH34^ZnR^ comprises six proteins that are encoded by Rmet_2229 up to Rmet_2234. More specifically, one periplasmic protein, one small integral membrane protein, two permeases, and two ATPases form the ABC transporter. The six genes encoding the ABC transporter resemble the glycerol ABC transporter operon of *R*. *leguminosarum* bv. *viciae* VF39, with for instance 63% amino acid identity between the periplasmic protein encoded by Rmet_2229 of CH34 and *glpV* of VF39 [58]. These similarities clearly indicate a role in glycerol uptake for this uncharacterized ABC-type transporter. In fact, addition of glycerol to the growth medium improved zinc resistance in a Rmet_2229-2234-dependent fashion, indicating a role for glycerol-mediated relief of repression of the ABC-type transporter. This observation therefore reveals that bacterial metal resistance can also depend on the exact carbon sources in the environment.

ABC transporters are found in all taxa and form one of the largest transporter superfamilies, containing both uptake and efflux transport systems. These transporters couple hydrolysis of ATP to the translocation of various substrates, ranging from single ions to entire protein toxins, across cell membranes [63]. The best-studied metal ABC transporter is the high-affinity Zn^2+^ uptake system encoded by the *znuABC* genes and regulated by Zur, which was initially reported in *E. coli* [20]. ZnuB is the membrane permease and ZnuC is the ATPase component of the transporter, whereas ZnuA is a soluble periplasmic metallochaperone that efficiently captures zinc in this cellular compartment and then delivers the metal to the transmembrane component of the transporter [64]. In contrast to eukaryotes, no examples of ABC transporters involved in metal export are known in bacteria. Nevertheless, ABC transporters that export substrates (which are still to be elucidated) to the periplasm could mediate the repair of metal-induced protein damage [65].

Based on its similarity to the glycerol utilization operon of *R*. *leguminosarum* bv. *viciae* VF39, the Rmet_2229-2234 transporter is likely an uptake system, and the exact mechanism underlying the observed zinc and cadmium resistance requires further study. Nevertheless, a few hypotheses could be formulated that might explain preventing excess zinc and cadmium in the cytoplasm. The imported substrates or the metabolism of these substrates might yield binding sites for metals [66,67]. However, no glycerol or sugars were added to the selective medium. Alternatively, the increased pool of periplasmic binding proteins could bind Zn^2+^ (Cd^2+^) and prevent entry into the cytoplasm. Lastly, released inorganic phosphate (P_i_) by ATP hydrolysis might bind excess Zn^2+^ (Cd^2+^), resulting in phosphate–zinc conjugates [68].

Finally, it is noteworthy that all of the mutations incurred by CH34^ZnR^ were caused by the transposition of different insertion sequences (more specifically IS*Rme5*, IS*Rme15*, and IS*1088*), underscoring the dynamic nature of these elements. Moreover, we have recently shown zinc-induced promoter activity for the transposases of IS*Rme5* and IS*1088* [69], indicating that IS dynamics can be boosted in times of stress to provide evolutionary escape routes for the host cell [69,70].

## 5. Conclusions

To conclude, we have demonstrated that derepression of the inconspicuous Rmet_2229-2234 ABC-type transporter (which is likely involved in glycerol uptake) can serve as an evolutionary road towards extreme zinc and cadmium resistance in *C. metallidurans* CH34. Moreover, Rmet_2229-2234-mediated protection against zinc can also become environmentally triggered in the presence of glycerol. Finally, our observations also underscore the importance of IS elements in the adaptive potential of *C. metallidurans* CH34.

## Figures and Tables

**Table 1:** Strains used in this study.

Strain	Genotype/Relevant Characteristic ^1^	References ^2^
*Cupriavidus metallidurans*		
CH34	pMOL28 pMOL30	[19]
CH34^ZnR^	pMOL28 pMOL30; increased resistance to Zn^2+^	This study
CH34 ∆*glpR*	*glpR* replaced by *tet*, Tc^R^	This study
CH34 ∆29-34	Rmet_2229 to Rmet_2234 replaced by *tet*, Tc^R^	This study
CH34^ZnR^ ∆29-34	Rmet_2229 to Rmet_2234 replaced by *tet*, Tc^R^	This study
*Escherichia coli*		
DG1	*mcrA* ∆(*mrr-hsdRMS-mcrBC*, modification-, restriction-) ϕ80*lacZ*∆M15 ∆*lacX74 recA1 araD139* ∆*(ara-leu)7697 galU galK rpsL endA1 nupG*	Eurogentec
HB101	F^-^ *mcrB mrr hsdS20(rB- mB-) recA13 leuB6 ara-14 proA2 lacY1 galK2 xyl-5 mtl-1 rpsL20 glnV44* λ-	Lab

^1^ Tc^R^: tetracycline resistant; ^2^ Eurogentec, Belgium; Lab: lab collection.

**Table 2:** Plasmids used in this study.

Strain	Genotype/Relevant Characteristic ^1^	References ^2^
pRK600	Helper plasmid; Cm^R^ *tra*	Lab
pBBR1MCS2	lacZα Km^R^ *ori* pBBR1 oriT	[45]
pBBR-*glpR*	*glpR* in pBBR1MCS2	This study
pBBR-*glpR*^R^	*glpR*::IS*1088* in pBBR1MCS2	This study
pACYC184	Cm^R^ Tc^R^ p15A ori	[46]
pK18mob	*lacZα* Km^R^ oriT oriV	[47]
p*glpR*	*glpR* in pK18mob, Km^R^	This study
pRmet_2229-34	Rmet_2229-2234 in pK18mob, Km^R^	This study
p*glpR::tet*	*glpR::tet* in pK18mob, Tc^R^ Km^R^	This study
pRmet_2229-34*::tet*	Rmet_2229-2234*::tet* in pK18mob, Tc^R^ Km^R^	This study

^1^ Cm: chloramphenicol; Km: kanamycin; Tc: tetracycline; ^R^resistant; ^2^ Lab: lab collection.

**Table 3:** Minimal inhibitory concentration (MIC, mM) of different metals determined in liquid MM284 medium for different *C. metallidurans* strains.

*C. metallidurans*	Zn^2+^	Ni^2+^	Co^2+^	Cd^2+^
CH34	12	2.5	5	1.5
CH34^ZnR^	24	2.5	5	3
CH34 ∆*glpR*	24	2.5	5	3
CH34 ∆29-34	12	2.5	5	1.5

**Table 4:** Mutations identified in CH34^ZnR.^

Gene	Protein	Inserted Element
Rmet_2146	ABC superfamily transporter subunit	IS*1088*
Rmet_2171	Conserved hypothetical protein	IS*Rme15*
Rmet_2235	DNA-binding transcriptional repressor	IS*1088*
Rmet_4452	Response regulator receiver domain protein (CheY-like)	IS*1088*
Rmet_4521	Transcriptional regulator	IS*Rme5*
Rmet_4574	Acyl-CoA dehydrogenase protein	IS*1088*
Rmet_5200	Putative glyoxalase/bleomycin resistance protein/dihydroxy-biphenyl dioxygenase	IS*1088*

## Data Availability

The full description of the microarray data has been deposited at the Gene Expression Omnibus website (http://www.ncbi.nlm.nih.gov/geo/) under accession number GSE156826. Sequencing data are available within the Sequencing Read Archive (SRA) of NCBI using the accession number PRJNA658861.

## Supplementary Materials

The following are available online at https://www.mdpi.com/2076-2607/9/2/309/s1, Figure S1: Zinc dose–response experiments with CH34 (blue) and CH34^ZnR^ (red). The abiotic (non-inoculated) control is shown in green. The average values of three independent experiments with standard deviations are shown. Figure S2: Relative fold change determined by quantitative real-time PCR (qRT-PCR) analysis of Rmet_2229 in CH34 DglpR and CH34^ZnR^. All data were normalized with 16S rRNA gene expression and given as relative to the parental CH34 strain. Figure S3: Transcription profile analysis of the Rmet_2234-*glpR* intergenic region from *C. metallidurans* CH34 (coordinates for the chromosomal region are shown at the bottom). Combined transcription start site (TSS) read counts of the three biological replicates are shown for positive (green) and negative (red) strands. The small black arrows indicate clearly identified primary and internal TSSs. The green arrow represents a re-annotated CDS. Data from [57], Table S1: Primers used in this study, Table S2: Differential gene expression analysis results.
